# Assessing White Wine Viscosity Variation Using Polarized Laser Speckle: A Promising Alternative to Wine Sensory Analysis

**DOI:** 10.3390/s17102340

**Published:** 2017-10-13

**Authors:** Christelle Abou Nader, Hadi Loutfi, Fabrice Pellen, Bernard Le Jeune, Guy Le Brun, Roger Lteif, Marie Abboud

**Affiliations:** 1 Physics Department, UR TVA, Faculty of Science, Saint Joseph University, B.P. 11-514-Riad El Solh, Beirut 1107 2050, Lebanon; hadi.loutfi@net.usj.edu.lb; 2 Laboratoire OPTIMAG (EA 938), Université de Bretagne Occidentale, 29238 Brest CEDEX 3, France; fabrice.pellen@univ-brest.fr (F.P.); bernard.lejeune@univ-brest.fr (B.L.J.); 3 Chemistry Department, UR TVA, Faculty of Science, Saint Joseph University, B.P. 11-514-Riad El Solh, Beirut 1107 2050, Lebanon; roger.lteif@usj.edu.lb

**Keywords:** speckle, diffusion, scattering, biological sensing

## Abstract

In this paper, we report measurements of wine viscosity, correlated to polarized laser speckle results. Experiments were performed on white wine samples produced with a single grape variety. Effects of the wine making cellar, the grape variety, and the vintage on wine Brix degree, alcohol content, viscosity, and speckle parameters are considered. We show that speckle parameters, namely, spatial contrast and speckle decorrelation time, as well as the inertia moment extracted from the temporal history speckle pattern, are mainly affected by the alcohol and sugar content and hence the wine viscosity. Principal component analysis revealed a high correlation between laser speckle results on the one hand and viscosity and Brix degree values on the other. As speckle analysis proved to be an efficient method of measuring the variation of the viscosity of white mono-variety wine, one can therefore consider it as an alternative method to wine sensory analysis.

## 1. Introduction

The wine industry is a major global economic hub. All stakeholders, including producers, need to improve controls of wine composition as well as its botanical and geographical origins [1,2], to improve the transparency of transactions and ultimately to prevent fraud. Even though novel methods for the assessment of wine quality and attributes are currently being considered [3,4,5,6], wine tasting through sensory analysis is still the most commonly used practice for the description of wine [7,8,9,10]. Two types of tasting are usually performed: (i) a horizontal one, where different bottles of wine produced within the same year (i.e., same vintage) are compared; and (ii) a vertical one, where a given wine is monitored while it undergoes aging.

The composition of the finished wine is a very complicated mixture of grape original components and those that were produced during the vinification of the wine, starting with fermentation as the first step, continuing along through the whole cellar operations, and ending up when the wine is aged in the bottle. Wine is composed of water (~84%), ethanol (~15%), and other minor components (~1%). Recently, it was shown that mouth feel, alcohol content, and other properties of wine can be determined using viscosity measurements [11,12,13,14]. In addition to ethanol, other components can have a significant effect on viscosity, mainly sugar and glycerol [15]. Mixtures of water and ethanol are more viscous than either liquid by itself, with the most viscous mixture occurring at 40% alcohol. At the typical 12% alcohol content of commercial wine and at 25 °C, the viscosity would be about 1.4 mPa.s vs. 0.89 mPa.s for pure water [15,16]. Sugar also contributes to the wine viscosity [11,12]. Glycerol is present at about one-tenth of the concentration of alcohol in most wines, this being higher for *botrytis*-affected grapes. While glycerol is very viscous by itself, it does not contribute significantly to the actual viscosity at the concentrations found in most wines [17]. Wine viscosity can thus be considered as a relevant criterion for the classification of wine, since viscosity values vary with parameters including grape variety, vintage, and temperature [11,12,13,14]. Information related to wine astringency, fluidity, dewatering, thickness, alcoholic, and sugar content can thus be obtained. Oenologists usually estimate the alcoholic and sugar content of wine during fermentation by measuring the density of the wine must, before the alcoholic fermentation and at the end of the fermentation process. Unfortunately, the predicted values of alcohol content often overestimate the real values in commercial wines [18]. One can also qualitatively assess alcohol and sugar content by twirling a glass of wine [19]. In addition to the release of the wine’s aromatic compounds in the air, twirling causes a portion of the wine to adhere to the walls of the glass. Evaporation-driven Marangoni flows near the meniscus of water–alcohol mixtures drive liquid upward, forming a thin liquid film, and a rim forms near the moving contact line. Eventually, the rim undergoes an instability, forming drops that roll back into bulk reservoir, forming the so-called “wine tears” or “wine legs.” When the wine is more viscous, the number of droplets increases, and the “tears” take longer time to form.

As the viscosity of the wine is of the order of only a few mPa.s, and low viscosity variations are measured when the terroir, grape variety, vintage, or wine temperature are considered [11,12,13,14], a sensitive and yet efficient method to determine this relative variation is thus needed. Since a polarized laser speckle method showed a high sensitivity for the measurement of small variations in viscosity [20], we employed it in our study to measure the viscosity of mono-variety white wines, and hence to characterize commercial wines. Speckle is produced when coherent light is scattered off an illuminated diffusing medium. Speckle pattern results from the interference of light scattered by the assembly of many optical inhomogeneities distributed randomly in space, such as particles in suspension [21]. In our work, we evaluate and compare various approaches of the polarized speckle field. Speckle patterns are analyzed by evaluating the spatial contrast [20,22], the temporal correlation coefficient [23], and the temporal history of the speckle pattern (THSP) [23,24,25].

In Section 2, we describe the wine samples and the speckle experimental setup. We also present the optical parameters extracted from the speckle patterns and other standard experiments. Experimental results are displayed in Section 3, along with the principal component analysis (PCA) that highlights the correlation between laser speckle results on one hand, and viscosity and Brix degree values on the other. Conclusions are drawn and future perspectives are considered in the last section.

## 2. Materials and Methods

### 2.1. White Wine Samples

A dozen bottles of commercial mono-variety white wine, i.e. each wine is produced using one grape variety, were chosen for this study. Several grape varieties (Chardonnay, Merwah, Pinot Gris and Sauvignon Blanc), vintages (2004, 2007, 2009, and 2012), and wine-making cellars (Château Bybline, Château Clos Saint Thomas, Château Florentine, Château Khoury, and Domaine Wardy) were considered. After the bottles of wine provided by the Lebanese wine making cellars were received, bottles were stored at room temperature (20 °C). For each wine bottle, and hence for each wine sample, we performed speckle, viscosity, alcohol content, and Brix degree measurements under 20 °C, within 4 to 5 h after the wine bottle was opened.

### 2.2. Speckle Experimental Setup and Parameters

**Figure 1:**
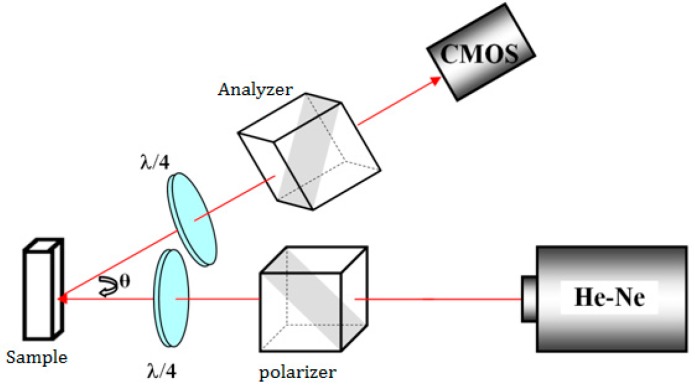
Schematic view of the speckle experiment setup. λ/4 is a quarter-wave plate.

The speckle experimental setup is illustrated in Figure 1. A volume of 0.3 mL of polystyrene microspheres (Polysciences Inc., Warrington, PA, USA), with an average diameter Ф = 0.22 µm and a refractive index of 1.59 suspended in water with an initial concentration of 0.00453 mg/mL, was added to a volume of 1.7 mL of white wine in a quartz cell. In fact, total attenuation coefficients (scattering and absorption coefficients), for all considered white wine samples, ranged from 10^−3^ to 2.10^−2^ mm^−1^ as measured using a Beer-Lambert experimental setup for collimated transmission [21]. Such scattering characteristics generated a backscattered signal that is too weak. In order to increase the scattered signal, and in order to allow the measurement of the speckle field with a sufficient signal-to-noise ratio, extrinsic scattering particles were added. The addition of microspheres to white wine made it possible to increase scattering and to obtain a total attenuation coefficient between 3 and 4 mm^−1^. This addition yielded an enhancement of the light diffused signal in low scattering media and hence allowed speckle measurements. This operation did not affect the viscosity of the wine sample. In fact, the volumic fraction of added microspheres in the total volume of the sample was approximately 10^−6^. Hence, one can reasonably consider that the viscosity of wine samples with added microspheres is equal to that of the wine samples [26]. A 15 mW He–Ne laser emitting at 632.8 nm, delivering a 1 mm wide linear polarized beam at $I_{0}/e^{2}$, where $I_{0}$ is the maximum laser intensity, with a coherence length of about 20 cm, illuminated the quartz cell. Backscattered light was captured by a high-speed recording Complementary Metal Oxide Semiconductor (CMOS) camera (MotionBLITZ EoSens mini, pixel size of 14 μm × 14 μm with a global electronic shutter) with an exposure time of 0.3 ms, appropriately chosen given the samples’ viscosity of only a few mPa.s, and a rate of 2130 fps. A polarizer and an analyzer were used in order to select the linear parallel or perpendicular scattered light with respect to the incident beam. Two quarter-wave plates were used to generate a circular polarization of incident light and to ensure a collection of circular backscattered light. The angle θ between the laser-sample axis and the sample-camera axis was equal to 20° in order to avoid specular reflection on the quartz cell. The speckle images were acquired using linear parallel (LP) and circular crossed (CC) light polarization configurations. The choice of these polarizations was made given the samples’ diffusing nature, in order to allow enough backscattered light intensity to reach the camera, which was set at a relatively short exposure time and in order to take into account both surface and volume diffusion, respectively [21,27].

Analysis of the speckle patterns, produced by each sample, was performed while considering a spatial and a temporal approach.

We undertake the spatial analysis by calculating the contrast C of a single speckle image [20,22]. The contrast is defined as the ratio between the intensity standard deviation σ and the mean intensity of the speckle pattern image, as follows:

(1) $$C=\frac{\sigma_{I}}{\langle I\rangle }\;=\frac{\sqrt{{\langle I\rangle }^{2}-{\langle I\rangle }^{2}}}{\langle I\rangle }.$$

For the temporal approach, speckle images were analyzed by computing the speckle intensity correlation on one hand [23], and extracting the temporal history of the speckle pattern (THSP) matrix on the other [24,25].

For each sample, we acquired a temporal series of speckle images with a frame rate of 2130 fps. We computed the speckle intensity correlation C(τ,Δx,Δy) with the mean of a cross correlation analysis between the first speckle image intensity $I\left(t_{0},x_{0},y_{0}\right)$ and the k-th speckle image intensity I(t,x,y) using

**Figure 2:**
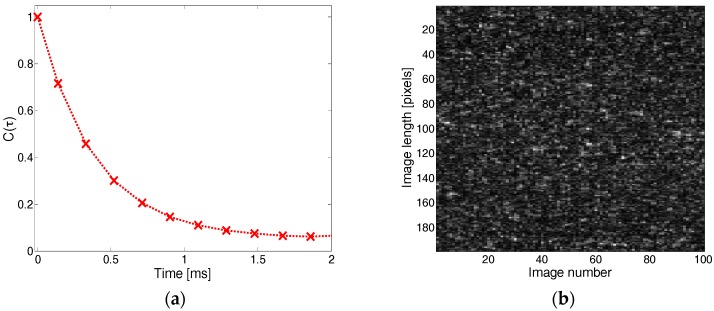
(**a**) Temporal correlation curve C(τ) and (**b**) the temporal history of the speckle pattern (THSP) image for a 1.7 mL white wine sample with 0.3 mL of added microspheres with a diameter of 0.22 µm.

(2) C(τ,Δx,Δy)=〈I(t0,x0,y0)I(t,x,y)〉−〈I(t0,x0,y0)I(t,x,y)〉{[〈I2(t0,x0,y0)〉−〈I(t0,x0,y0)〉2][〈I2(t,x,y)〉−〈I(t,x,y)〉2]}12

where $\tau \;=\;t\;-{\;t}_{0}$ is the time, $\Delta x\;=\;x-\;x_{0}$, $\Delta y\;=\;y-\;y_{0}$, (Δx,Δy)=(mΔr,nΔr), m and n are the pixel positions in the image, and Δr is the pixel size [23]. An example of the temporal correlation curve is plotted in Figure 2a as a function of time, giving an idea of the analyzed sample activity evolution. Taking into account the analyzed light polarizations (linear parallel and cross circular) in a backscattered geometry [27], as well as the low scattering nature of our samples [20], we regard the collected speckle fields to be mainly generated by photons that have undergone a simple diffusion. Therefore, an exponential fit of the correlation curve $ae^{b\tau }+d$, where $b=-1/\tau_{c}$, and d=1−a, allows for an estimation of the speckle decorrelation time constant $\tau_{c}$. As shown in previous studies [20,22], the speckle decorrelation time constant $\tau_{c}$ is linearly correlated to the samples’ viscosity.

Furthermore, as part of the temporal analysis, the THSP matrix was extracted from a series of speckle images. After recording N continuous speckle images, the middle column is chosen from each one. These columns are then positioned side by side, according to their chronological order, to create a new image called the THSP. The objective of the THSP is to study intensity variations in a horizontal direction. As each row of the matrix represented the intensity variation of a speckle column with time, the THSP could monitor horizontal time fluctuations [24]. If a slow activity is present in the diffusing sample, the speckle temporal variations associated with the sample are slow and the THSP shows stretching shapes. If the phenomenon is very dynamic and the grain size is almost equal to camera pixel size, the THSP is similar to an ordinary speckle image. An example of a THSP image for a white wine sample is illustrated in Figure 2b. The THSP allows us to calculate the inertia moment IM defined by

(3) $$\mathrm{IM}=\sum M_{i,j}{\left(i-j\right)}^{2}$$

where $M_{i,j}$ is the (*i, j*) pixel gray value of the normalized co-occurrence matrix (MCOM) [24,28]. This value indicates the number of occurrences of a certain intensity value *i*, followed instantly by an intensity value *j*. IM is useful for estimating a sample’s total activity. When the activity of a sample decreases, its variations become slower, and IM thus decreases [23,28,29].

As samples scattering and absorption properties might influence the parameters calculated via speckle images [20,30], scattering and absorption coefficients were measured for all wine samples considered in our study. The results show almost the same scattering and absorption coefficients for all samples, allowing us to attribute changes in optical parameters exclusively to changes in the samples’ viscosity.

### 2.3. Viscosity Measurement

**Figure 3:**
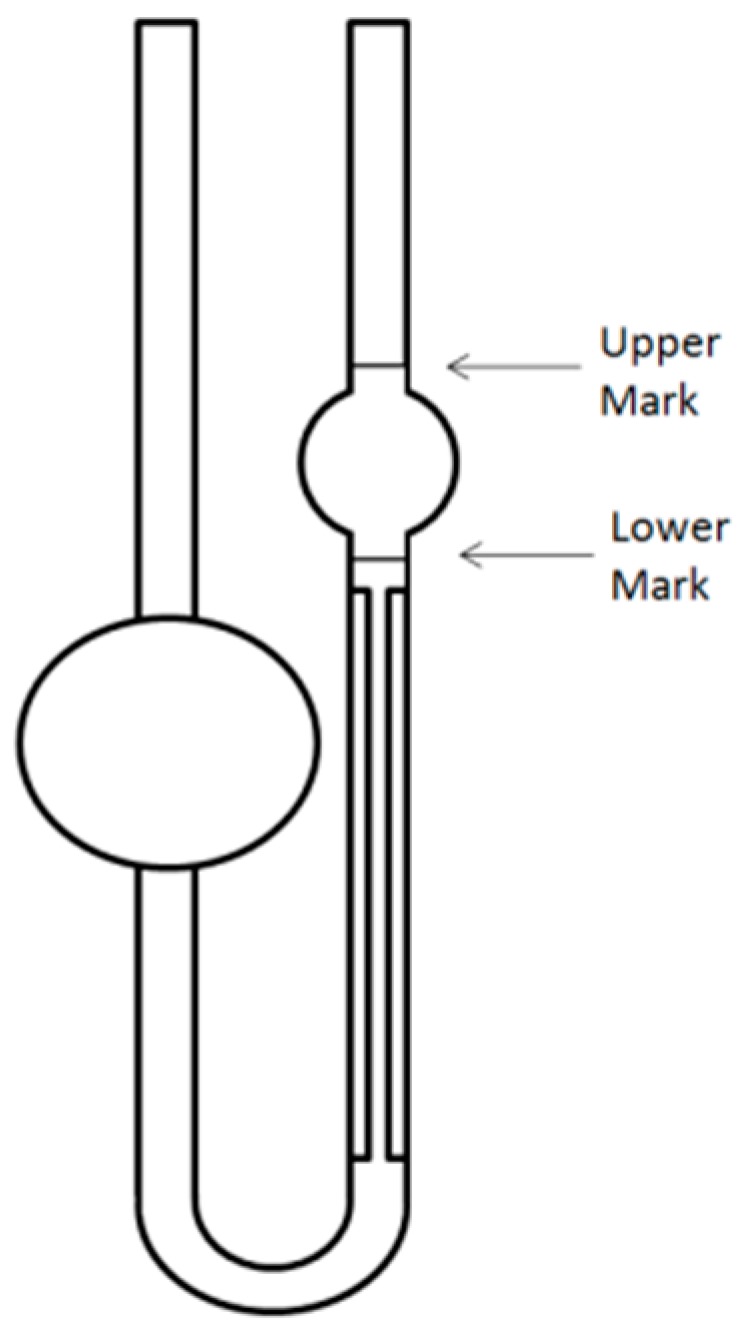
Sketch of an Oswald viscometer showing the upper and the lower marks.

The viscosity of the wine samples was measured through a calibrated glass capillary Ostwald viscometer. Liquid, with no-added microspheres, was drawn into the upper bulb via suction, and then allowed to flow down through the capillary into the lower bulb. The time δt taken for the level of the liquid to pass between two marks, one above and one below the upper bulb (see Figure 3), and measured with a stopwatch, is proportional to the viscosity η. Consequently, the viscosity η is deduced using the following formula:

(4) η = K δt ρ

where δt is the measured time, K is an instrumental constant, and ρ is the liquid density. This measurement was repeated four times for each of the considered samples, and the average value is given in the results section.

### 2.4. Alcohol Content and Brix Degree Measurements

Alcohol content was determined using a density meter (Anton-Paar BMA 4500M) after the wine was distilled. Values correspond to the dissolved alcohol in wine.

The Brix degree was measured using a portable refractometer (FG-103) that permits measurements in the range 0–32%.

To account for repeatability, all measurements were performed four times, and mean values are given in the results section.

## 3. Results and Discussion

### 3.1. Viscosity Measurement Using Speckle

We adopted various approaches of the polarized speckle field for the optical measurements in the wine samples with different grape varieties, vintages, and wine making cellars. Both speckle parameters, the spatial contrast C, and the decorrelation time $\tau_{c}$ in LP and CC polarization configurations, were considered for the viscosity variations of the different samples of white wine.

**Figure 4:**
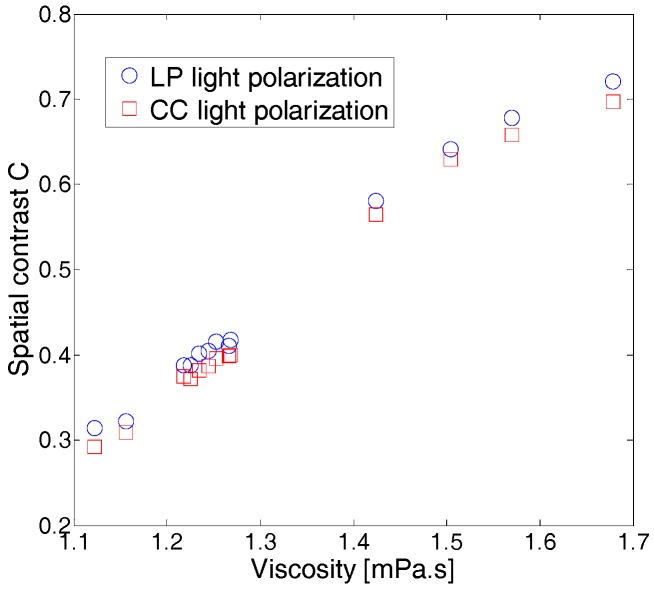
Variation of the speckle spatial contrast C as a function of white wine viscosity. Standard deviations are approximately ±0.001 mPa.s for the viscosity, and ±0.002 for the contrast C. Error bars are not displayed on the figures for the sake of clarity.

Figure 4 shows the evolution of the contrast C as a function of the measured viscosity. For both of the considered light polarization configurations (LP and CC), we found that the contrast values increases when the viscosity of the wine increases. As shown in other studies, when the sample viscosity increases, the Brownian motion of the suspended particles in the sample shows down, yielding a decrease in the speckle intensity fluctuations. As a result, the contrast C increases. This behavior has been previously observed, and the contrast of the speckle images has been correlated to samples viscosities [20]. Furthermore, we found that contrast values in the LP light configuration are slightly larger than those obtained in the CC light polarization configuration. This is consistent with the polarization memory effect of backscattered light by a turbid medium, which depends on the size of the scatterers [27,31]. In the case of small particles with respect to an optical wavelength, as in our situation, scattering is equally likely in all directions. Linear polarization is hence maintained for longer than circular one [27]. Backscattering does not affect the linear character of linearly polarized light, but it reverses the helicity of circularly polarized light and randomizes it more rapidly. Contrarily, when the scattering medium contains large particles with respect to optical wavelength, it has been shown that for pathlengths of the order of a transport mean free path, linearly polarized light is randomized, whereas circularly polarized light preserves its original polarization [32], yielding better speckle image contrast when circularly polarized light is used, as reported in a previous study [33].

**Figure 5:**
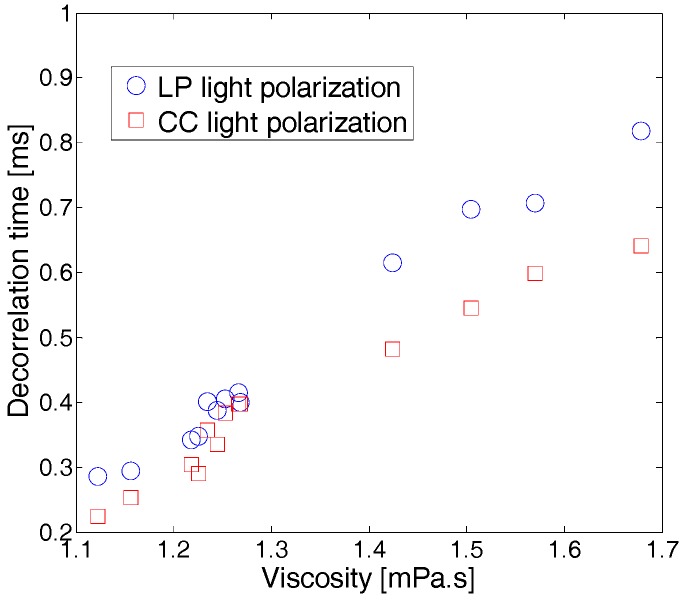
Variation of the speckle decorrelation time $\tau_{c}$ as a function of white wine viscosity. Symbols correspond to experimental values and lines correspond to linear fits. Standard deviations are approximately ±0.001 mPa.s for the viscosity, and ±0.006 ms for the decorrelation time τ. Error bars are not displayed on the figures for the sake of clarity.

For the temporal approach, the decorrelation time $\tau_{c}$ is extracted using an exponential fit of the correlation coefficient curve as already mentioned. In fact, $\tau_{c}$ is directly proportional to the stirring motion of the particles in a solution [20,31,34]. In addition, it was proven that the speckle decorrelation time increases linearly with the viscosity of the solution [20]. As presented in Figure 5, results similar to those obtained using the spatial approach were also found for different wine samples having different viscosities: as the wine viscosity increases, speckle decorrelation time also increases. Furthermore, $\tau_{c}$ values in the LP light configuration are slightly larger than the results obtained in CC light polarization configuration. Here, and similarly to the spatial contrast approach, since linear polarization is preserved for longer paths than the circular one because of the small dimension of optical scatterers, larger decorrelation times are measured when linearly polarized light is used [27,32].

We discuss in the following the efficiencies of both procedures, particularly in terms of sensitivity and the duration of the measurement. When wine viscosity covers the range [1.1, 1.7] mPa.s, and therefore over a relative variation of about 55%, the contrast shows an increase by a factor 2.3 in both the LP and the CC polarization configurations. Meanwhile, the decorrelation time varies by a factor of 3, ranging from 0.22 to 0.65 ms and from 0.28 to 0.82 ms in CC and LP polarization configurations, respectively. This shows the efficiency and sensitivity of the temporal approach in detecting viscosity variations, as small as they may be. From another perspective, if one compares the duration of each type of measurement, one can clearly see that the spatial approach presents a substantial advantage with respect to the speckle temporal approach and viscosity measurement using Ostwald viscometer. In fact, the latter methods are time-consuming, whereas the spatial contrast approach can be performed in less than 1 s since it relies on the acquisition of a single speckle image, allowing for the evaluation of the viscosity value at a glance [20].

### 3.2. The Effect of the Wine Making Cellar on Wine Viscosity

In order to evaluate the effect of the winery domain on white wine characteristics, we considered samples obtained from different wine making cellars, Château Florentine, Domaine Wardy, and Château Clos Saint Thomas, with identical vintage (2012) and grape variety (Chardonnay).

Results indicate that the spatial contrast C and the decorrelation time $\tau_{c}$ values for LP and CC polarization configurations are substantially identical with a standard deviation in the range of ±0.002 for C and ±0.006 ms for $\tau_{c}$. In addition, the three samples have exactly the same Brix degree value (6.90), while viscosity and alcohol content are identical with standard deviations in the range of ±0.001 mPa.s and 0.3%, respectively. We can deduce that the viscosity, the alcohol content, and the Brix degree are not affected by the wine making cellar as long as the same grape variety and vintage are considered. This may be justified by the fact that similar production processes are applied while producing commercial wines.

### 3.3. The Effect of the Grape Variety on Wine Viscosity

As the wine making cellar has little effect on wine viscosity, after setting 2012 as the year of production, we considered four different grape varieties, Sauvignon Blanc, Merwah, Chardonnay, and Pinot Gris, to determine their effect on wine viscosity.

As shown in Table 1a, the viscosity changes from one grape variety to another. This variation is consistent with analysis carried out in a previous study on French mono-variety wines [14]. In fact, each grape variety has its own features during fermentation. This behavior is demonstrated by the fluctuation in Brix degree values and alcohol content [11,12,13].

In addition to the measurements of the alcohol content and Brix degree, the speckle analysis results also show a variation of the contrast C, the speckle decorrelation time $\tau_{c}$, and the inertia moment IM values (see results displayed in Table 1b). Since wine samples present low scattering and absorption coefficients, we can attribute changes in optical parameters to changes in the samples’ viscosity. In our results, while wine viscosity increases from 1.2254 to 1.5046 mPa.s., the contrast of the speckle images rises significantly and the decorrelation time increases, as microspheres added to the wine tend to move slower [20,30]. Moreover, the results show a decrease in the values of IM. In fact, this speckle parameter can be linked to the sample’s total speckle activity. When the viscosity of the sample becomes higher, the Brownian motion of microbeads added to the wine is slowed down, resulting in lower IM values [23,28,29].

Plotting the variation of each parameter as a function of viscosity would lead to a high number of similar graphs that would be too cumbersome if shown at once. Thus, for the sake of clarity, and in order to establish all possible correlations between experimental data, we used principal component analysis (PCA) as a statistical method. PCA helps reduce the number of variables and spots a relationship between them [35]. The calculus was made using the XLSTAT program. The speckle parameters (the contrast C, the inertia moment IM, and the decorrelation time $\tau_{c}$) in both LP and CC light polarization configurations, as well as viscosity, Brix degree, and alcohol content were taken into account.

We present in Table 2 the correlation between the variables. A correlation is found between the viscosity and the Brix degree, with a Pearson correlation coefficient equal to 0.942. This correlation shows a connection between the viscosity and the sugar concentration in the wine. The correlations between inertia moment values and all other parameters, when taken one by one, are negative. This simply indicates that these parameters vary in opposite directions, while being highly correlated. Finally, high correlation values (with values of Pearson correlation coefficient ranging between 0.946 and 0.997) are obtained between viscosity and all speckle parameters (contrast and decorrelation time in LP and CC light polarization configurations).

**Figure 6:**
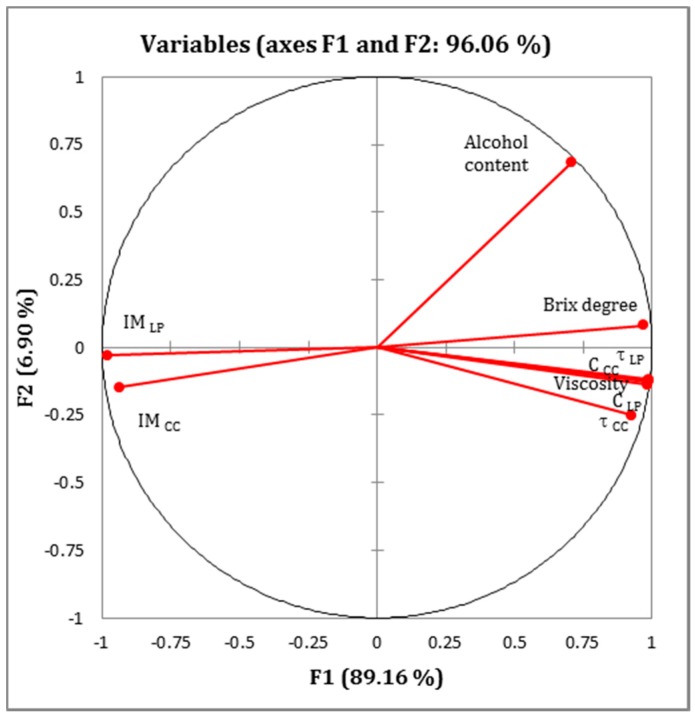
Pearson correlation circle representing the variables projected on the first principal component F1 and the second principal component F2 for all wine samples.

The Pearson circle contains the different variables used in this study (Figure 6). The statistical calculation shows a first principal component F1 at 89.16% and a second principal component F2 at 6.90%. The F1 and F2 interrelation was set at 96.06%. In addition, the decorrelation time $\tau_{c}$ and the spatial contrast C in the two light polarization configurations (LP and CC) are highly correlated to the first component axis F1. However, the inertia moment IM is negatively correlated to $\tau_{c}$ and C, and hence F1. Indeed, when $\tau_{c}$ and C values increase because of slower dynamics in the sample, IM values decrease as previously stated. Finally, viscosity and Brix degree values are strongly correlated with F1, whereas alcohol content is distant from both F1 and F2. In fact, the Brix degree reflects the amount of residual sugars in the wine. When the latter increases, the viscosity also increases. However, the dissolved alcohol affects the wine viscosity to a lesser extent. This is in perfect agreement with previously reported results in white wine, where, across a large range of sugar concentrations, it was revealed that sugar drastically influences the viscosity, whereas ethanol has only a moderate effect [11,12].

## 4. Conclusions

In this paper, we show that it is possible to estimate wine viscosity by using the polarized laser speckle method and by calculating either the spatial contrast of a speckle pattern, either the speckle decorrelation time between a series of speckle patterns. This study allowed us to conclude that wines produced from different wine-making cellars have the same viscosity and alcoholic content. This may be justified by the fact that similar production processes are applied while producing commercial wines. However, the variation of the grape variety has an important influence on the white wine viscosity. This variation is in agreement with previous studies on French white, red, and rosé wines [14]. Moreover, speckle spatial and temporal parameters proved to be sensitive to wine viscosity variations, as we showed that the spatial contrast and the speckle decorrelation time increase with the viscosity of the wine samples. Optical parameters (spatial contrast C, speckle decorrelation time $\tau_{c}$, and inertia moment IM) were correlated to other parameters issued from standard measurements (viscosity, alcoholic content, and Brix degree) by means of a statistical approach. PCA showed high correlation levels between the different speckle parameters on one hand and the wine viscosity and Brix degree that can be linked to the sugar content on the other.

In the near future, we consider applying this study to red and rosé wines. Moreover, we envisage an extension of our study to the case of wine samples issued from a mix of different grape varieties. In the long term, we are confident that the polarized laser speckle method can be used to characterize grape varieties, to date wine, and even to detect wine alteration and, eventually, fraud. The polarized laser speckle method could also constitute an alternative to commonly used methods of testing wines, such as sensorial analysis, even though not all tasting-related aspects would not be considered.

Since causal links exist between the viscosity and the microstructure of biological fluids, one can similarly tackle other food-related applications within inspection activities [36]. For instance, in the quality control sector, the measurement of viscous properties can serve as an indirect tool for defining product consistency and the quality of various foodstuffs such as milk [37], ketchup [38], oil [39], and honey [40].

## Figures and Tables

**Table 1:** Results of the effect of the grape variety on white wine (**a**) viscosity, Brix degree, and alcohol content and (**b**) speckle results in linear parallel (LP) and circular crossed (CC) light polarization configurations. Standard deviations are approximately ±0.001 mPa.s for the viscosity, ±0.01 for the Brix degree, ±0.02% for the alcohol content, ±0.002 for the contrast C, ±0.006 ms for the decorrelation time $\tau_{C}$, and ±170 for the inertia moment IM.

Winery	Clos Saint Thomas	Bybline	Wardy	Florentine	Clos Saint Thomas	Khoury
(**a**)						
grape variety	Sauvignon Blanc	Merwah	Chardonnay	Chardonnay	Chardonnay	Pinot Gris
viscosity (mPa.s)	1.225	1.253	1.266	1.268	1.262	1.505
Brix (°Brix)	6.95	6.50	6.90	6.90	6.90	9.50
alcohol content (%)	13.75	12.06	12.68	13.10	12.62	13.87
(**b**)						
grape variety	Sauvignon Blanc	Merwah	Chardonnay	Chardonnay	Chardonnay	Pinot Gris
$C_{LP}$	0.3874	0.4154	0.4108	0.4170	0.4127	0.6408
$C_{CC}$	0.3715	0.3954	0.3987	0.3999	0.3976	0.6289
$\tau_{CLP}$ (ms)	0.3470	0.4050	0.4148	0.4000	0.4113	0.6973
$\tau_{CCC}$ (ms)	0.2895	0.3833	0.3967	0.3968	0.3973	0.5454
$IM_{LP}$	4333	3970	3876	3877	3860	1842
$IM_{CC}$	3549	3499	3281	3229	3269	1371

**Table 2:** Pearson correlation coefficient matrix between values of speckle parameters, viscosity, Brix degree, and alcohol content measurements for all wine samples.

Variable	Viscosity	Brix Degree	Alcohol Content	C _LP_	C _CC_	τ _C LP_	τ _C CC_	IM _LP_	IM _CC_
Viscosity	**1**	_	_	_	_	_	_	_	_
Brix degree	0.942	**1**	_	_	_	_	_	_	_
Alcohol content	0.618	0.718	**1**	_	_	_	_	_	_
C _LP_	0.996	0.958	0.625	**1**	_	_	_	_	_
C _CC_	0.997	0.959	0.618	1	**1**	_	_	_	_
τ _C LP_	0.994	0.942	0.623	0.994	0.992	**1**	_	_	_
τ _C CC_	0.946	0.821	0.52	0.93	0.928	0.955	**1**	_	_
IM _LP_	−0.935	−0.946	−0.685	−0.947	−0.941	−0.962	−0.897	**1**	_
IM _CC_	−0.865	−0.917	−0.713	−0.88	−0.874	−0.9	−0.813	0.982	**1**

## References

[1] Van Leeuwen C., Friant P., Choné X., Tregoat O., Koundouras S., Dubourdieu D. (2004). Influence of climate, soil, and cultivar on terroir. Am. J. Enol. Vitic..

[2] Bejjani J., Balaban M., Rizk T. (2014). A sharper characterization of the geographical origin of Lebanese wines by a new interpretation of the hydrogen isotope ratios of ethanol. Food Chem..

[3] Chung S., San Park T., Hyun Park S., Yong Kim J., Park S., Son D., Min Bae Y., In Cho S. (2015). Colorimetric sensor array for white wine tasting. Sensors.

[4] Di Gennaro S.F., Matese A., Mancin M., Primicerio J., Palliotti A. (2014). An open-source and low-cost monitoring system for precision enology. Sensors.

[5] Aguilera T., Lozano J., Paredes J.A., Alvarez F.J., Suarez J.I. (2012). Electronic nose based on independent component analysis combines with partial least squares and artificial neural networks for wine prediction. Sensors.

[6] Gutierrez M., Llobera A., Ipatov A., Vila-Planas J., Minguez A., Demming A., Buttgenbach S., Capdevila F., Domingo C., Jimenez-Jorquera C. (2011). Application of an E-tongue to the analysis of monovarietal and blends of white wines. Sensors.

[7] Murray J.M., Delahunty C.M., Baxter I.A. (2001). Descriptive sensory analysis: Past, present and future. Food Res. Int..

[8] Legin A., Rudnitskaya A., Lvova L., Vlasov Yu., Di Natale C., D’Amico A. (2003). Evaluation of Italian wine by the electronic tongue: Recognition, quantitative analysis and correlation with human sensory perception. Anal. Chim. Acta.

[9] Cliff M.A., King M.C., Schlosser J. (2007). Anthocyanin, phenolic composition, colour measurement and sensory analysis of BC commercial red wines. Food Res. Int..

[10] Lima Ferreira M., Amaral B., Salagoïty M.H., Lagrèze C., de Revel G., Médina B. (2016). Document sur L’analyse Sensorielle du vin. Partie I: Conditions Générales Pour la Réalisation de Tests D’analyse Sensorielle.

[11] Burns D.J.W., Noble A.C. (1985). Evaluation of the separate contributions of viscosity and sweetness of sucrose to perceived viscosity, sweetness and bitterness of vermouth. J. Texture Stud..

[12] Nurgel C., Pickering G. (2005). Contribution of glycerol, ethanol and sugar to the perception of viscosity and density elicited by model white wines. J. Texture Stud..

[13] Yanniotis S., Kotseridis G., Orfanidou A., Petraki A. (2006). Effect of ethanol, dry extract and glycerol on the viscosity of wines. J. Food Eng..

[14] Siret R., Madieta E., Symonaux R., Jourjon F. Mesures rhéologiques de la texture et de la viscosité des vins. Corrélations avec l’analyse sensorielle. Proceedings of the 31st World Congress of Vine and Wine, 6th General Assembly of the O.I.V..

[15] Margalit Y. (2016). Concepts in Wine Chemistry.

[16] Pickering G.J., Heatherbell D.A., Vanhanen L.P., Barnes M.F. (1998). The effect of ethanol concentration on the temporal perception of viscosity and density in white wine. Am. J. Enol. Vitic..

[17] Noble A.C., Bursick G.F. (1984). The contribution of glycerol to perceived viscosity and sweetness in white wine. Am. J. Enol. Vitic..

[18] Ribéreau-Gayon P., Dubourdieu D., Donèche B., Lonvaud A. (2006). Handbook of Enology Volume 1 The Microbiology of Wine and Vinifications.

[19] Venerus D.C., Simavilla D.N. (2015). Tears of wine: New insights on an old phenomenon. Sci. Rep..

[20] Abou Nader C., Pellen F., Roquefort P., Aubry T., Le Jeune B., Le Brun G., Abboud M. (2016). Evaluation of low viscosity variations in fluids using temporal and spatial analysis of the speckle pattern. Opt. Lett..

[21] Abou Nader C., Nassif R., Pellen F., Le Jeune B., Le Brun G., Abboud M. (2015). Influence of size, proportion, and absorption coefficient of spherical scatterers on the degree of light polarization and the grain size of speckle pattern. Appl. Opt..

[22] Fercher A.F., Briers J.D. (1981). Flow visualisation by means of single-exposure speckle photography. Opt. Commun..

[23] Nassif R., Abou Nader C., Pellen F., Le Brun G., Abboud M., Le Jeune B. (2013). Retrieving controlled motion parameters using two speckle pattern analysis techniques: Spatiotemporal correlation and the temporal history speckle pattern. Appl. Opt..

[24] Haralick R.M., Shanmugam K., Dinstein I. (1973). Textural features for image classification. IEEE Trans. Syst. Man Cybern..

[25] Oulamara A., Tribillon G., Duvernoy J. (1989). Biological activity measurements on botanical specimen surfaces using a temporal decorrelation effect of laser speckle. J. Mod. Opt..

[26] Einstein A. (1906). Eine neue Bestimmung der Moleküldimensionen. Ann. Phys..

[27] Morgan S.P., Ridgway M.E. (2000). Polarization properties of light backscattered from two layer scattering medium. Opt. Exp..

[28] Arizaga R., Trivi M., Rabal H. (1999). Speckle time evolution characterization by the co-occurrence matrix analysis. Opt. Laser Technol..

[29] Nassif R., Abou Nader C., Afif C., Pellen F., Le Brun G., Le Jeune B., Abboud M. (2014). Detection of golden apples’ climacteric peak by laser biospeckle measurements. Appl. Opt..

[30] Hajjarian Z., Nadkarni S.K. (2014). Correction of optical absorption and scattering variations in laser speckle rheology measurements. Opt. Exp..

[31] Piederriere Y., Boulevert F., Cariou J., Guern Y., Le Brun G. (2005). Backscattered speckles size as a function of polarization: Influence of particle-size and concentration. Opt. Exp..

[32] MacKintosh F.C., Zhu J.X., Pine D.J., Weitz D.A. (1989). Polarization memory of multiply scattered light. Phys. Rev. B.

[33] Ni X., Alfano R.R. (2004). Time-resolved backscattering of circularly and linearly polarized light in a turbid medium. Opt. Lett..

[34] Potanin A.A. (1993). On the self-consistent calculations of the viscosity of colloidal dispersions. J. Colloid Interface Sci..

[35] Abdi H., Hervé L. (2010). Principal component analysis. WIREs Comput. Stat..

[36] Brown A. (2015). Understanding Food: Principles and Preparation.

[37] Fernández-Martín F. (1972). Influence of temperature and composition on some physical properties of milk and milk concentrates. II. Viscosity. J. Dairy Res..

[38] McCarthy K.L., McCarthy M.J. (2009). Relationship between in-line viscosity and Bostwick measurement during ketchup production. J. Food Sci..

[39] Bonnet J.P., Devesvre L., Artaud J., Moulin P. (2011). Dynamic viscosity of olive oil as a function of composition and temperature: A first approach. Eur. J. Lipid Sci. Technol..

[40] El Sohaimya S.A., Masryb S.H.D., Shehataa M.G. (2015). Physicochemical characteristics of honey from different origins. Ann. Agric. Sci..

